# Supplementary material for: Wound Healing Potential of an Oleoresin Essential Oil Chemotype from Canarium schweinfurthii Engl
Source: Molecules. 2022 Nov 17;27(22):7966. doi: 10.3390/molecules27227966 (PMC9697663; doi:10.3390/molecules27227966)
Supplement: Supplementary file 1 [file molecules-27-07966-s001.zip › molecules-2028419-supplementary.pdf]

Supplementary Materials for

Wound Healing Potential of an Oleoresin Essential Oil Chemotype from *Canarium schweinfurthii* Engl.

Michel Bonnard *, Enzo Martin and Isabelle Parrot *

* Correspondence: michel.bonnard@umontpellier.fr (M.B.); isabelle.parrot-smietana@umontpellier.fr (I.P.)

Table S1. Chemical composition of EO (% Area, non-polar phase) obtained from oleoresins of *C. schweinfurthii* collected in Gabon (Ntoum region, trees #1-#4).

No.	Compounds	Composition (% Area)			
		Tree #1	Tree #2	Tree #3	Tree #4
1	α-thujene	0.28	0.13	0.67	0.09
2	α-pinene	5.69	21.01	2.04	5.91
3	camphene	0.06	0.13	0.04	nd
4	sabinene	8.36	4.22	20.13	1.58
5	β-pinene	3.41	1.42	0.96	0.46
6	2-menthene	1.87	nd	nd	nd
7	α-phellandrene	19.04	20.49	4.71	3.20
8	δ^3-carene	0.19	nd	nd	nd
9	α-terpinene	2.92	1.14	1.16	0.03
10	*p*-cymene	9.26	6.51	14.90	10.65
11	limonene	10.70	25.22	17.36	48.36
12	β-phellandrene	2.20	nd	nd	nd
13	eucalyptol	0.12	3.66	1.96	3.07
14	γ-terpinene	1.68	0.76	1.93	0.06
15	4-thujanol	0.09	nd	nd	nd
16	terpinolene	32.17	1.04	16.44	0.20
17	*p*-cymenene	0.25	nd	nd	nd
18	terpinen-4-ol	0.55	0.53	0.20	0.44
19	*p*-cymen-8-ol	0.92	nd	nd	nd
20	α-terpineol	0.26	9.09	4.71	24.31
21	*cis*-thujanol	nd	0.99	0.80	0.23
22	β-caryophyllene	nd	0.66	nd	nd
23	*trans*-β-ocimene	nd	0.21	0.14	0.25
24	α-dihydroterpineol	nd	0.21	nd	nd
25	*trans*-thujanol	nd	0.11	0.26	0.10
26	camphor	nd	0.09	nd	nd
27	*cis*-2-menthenol	nd	0.08	0.26	nd
28	humulene	nd	0.07	nd	nd
29	*trans*-2-menthenol	nd	0.06	0.04	nd
30	cyperene	nd	0.05	nd	nd
31	pipertone	nd	0.04	nd	0.06
32	2-(4-methylphenyl)propan-2-ol	nd	nd	2.92	nd

Tables S2. *In chemico* assessment of skin sensitization (raw data)

	**Negative control**		**Positive control 1**		**Positive control 2**		**EO**	
Cysteine peptide assays	(-)peptide	(+)peptide	(-)peptide	(+)peptide	(-)peptide	(+)peptide	(-)peptide	(+)peptide
FI_1	2196	32857	1048	9535	2167	14238	2252	17223
FI_2	2268	32878	1041	9755	2145	14224	2201	16943
FI_3	2269	32742	1053	9637	2152	13763	2234	17005
Mean	2244	32826	1047	9642	2155	14075	2229	17057
St. Dev.	42	73	6	110	11	270	26	147
$FI_{corr} = [FI_{(+)} – FI_{(-)}]$	FI_{corr}	30581	FI_{corr}	8595	FI_{corr}	11920	FI_{corr}	14828
Peptide depletion	$PD_{Cyst.}$	-	$PD_{Cyst.}$	71.9%	$PD_{Cyst.}$	61.0%	$PD_{Cyst.}$	51.5%

Calibration curve		
Cyst.	μM	FI
Std 0	0	2199
Std 1	6.25	3584
Std 2	12.5	4735
Std 3	25	6491
Std 4	50	10171
Std 5	100	17946
Std 6	200	32831

	**Negative control**		**Positive control 1**		**Positive control 2**		**EO**	
Lysine peptide assays	(-)peptide	(+)peptide	(-)peptide	(+)peptide	(-)peptide	(+)peptide	(-)peptide	(+)peptide
FI_1	1684	30925	729	2972	1568	18024	1624	28819
FI_2	1687	31465	718	3040	1587	17586	1581	28231
FI_3	1673	31726	762	3003	1582	17834	1553	27930
Mean	1681	31372	736	3005	1579	17815	1586	28327
St. Dev.	7	409	23	34	10	220	36	452
$FI_{corr} = [FI_{(+)} – FI_{(-)}]$	FI_{corr}	29691	FI_{corr}	2269	FI_{corr}	16236	FI_{corr}	26741
Peptide depletion	$PD_{Lys.}$	-	$PD_{Lys.}$	92.4%	$PD_{Lys.}$	45.3%	$PD_{Lys.}$	9.9%

Calibration curve		
Lys.	μM	FI
Std 0	0	1627.5
Std 1	3.125	2388.5
Std 2	6.25	3413
Std 3	12.5	5515.5
Std 4	25	9171
Std 5	50	16819
Std 6	100	31330.5

Tables S3. *In vitro* assessment of skin sensitization (raw data)

Cytotoxicity assay on THP1 cells – 24hrs treatment										
EO (mg/mL)	Control	0.00005	0.0001	0.0005	0.001	0.005	0.01	0.05	0.1	0.2
$FI\text{-}1_{corr}$	40872	41024	40080	39830	39482	38235	37337	34986	27907	7315
$FI\text{-}2_{corr}$	41096	40761	40382	39868	39139	38732	37069	34504	27541	7440
$FI\text{-}3_{corr}$	40930	40818	40546	39874	39436	38591	37192	35108	28019	7338
Mean	40966	40868	40336	39858	39353	38520	37200	34866	27823	7365
St. Dev.	116	138	236	24	186	256	134	319	250	67
Viability (%)	100	100	98	97	96	94	91	85	68	18
Cytotoxicity (%)	-	0	2	3	4	6	9	15	32	82

Cytotoxicity assay on THP1 cells – 24hrs treatment										
EO (mg/mL)	Control	0.010	0.013	0.018	0.024	0.032	0.042	0.056	0.075	0.1
$FI\text{-}1_{corr}$	40589	38788	38263	37665	37640	36710	35768	34033	31007	25738
$FI\text{-}2_{corr}$	40554	38097	37858	37181	36290	35967	35671	34131	31412	25753
$FI\text{-}3_{corr}$	40768	38302	37605	37697	37356	35681	35681	34210	31108	25539
$FI\text{-}4_{corr}$	41098	37960	37963	37841	36966	36078	36078	33929	31238	25999
Mean	40752	38287	37922	37596	37063	36634	35799	34076	31191	25757
St. Dev.	249	363	272	287	585	518	191	122	175	188
Viability (%)	100	94	93	92	91	90	88	84	77	63
Cytotoxicity (%)	-	6	7	8	9	10	12	16	23	37

CD86 expression assay on THP1 cells – 24hrs treatment

	Control	$Contr._{EtOH}$	PC	EO mg/mL					
CD86 assay	-	EtOH (0.2%)	DCNB 5µg/mL	0.036	0.043	0.052	0.063	0.075	0.090
$OD_{mes.1}$	0.226	0.236	0.761	0.358	0.417	0.543	0.598	0.614	0.627
$OD_{mes.2}$	0.210	0.233	0.743	0.340	0.440	0.558	0.587	0.608	0.643
$OD_{mes.3}$	0.225	0.239	0.792	0.343	0.410	0.567	0.608	0.632	0.625
Mean	0.220	0.236	0.766	0.347	0.422	0.556	0.598	0.618	0.632
St. Dev.	0.009	0.003	0.025	0.010	0.016	0.012	0.011	0.012	0.010
OD_{CD86}									
Mean	0.424	0.485	2.604	0.930	1.232	1.767	1.933	2.014	2.069
St. Dev.	0.036	0.013	0.100	0.038	0.062	0.049	0.042	0.049	0.039
Resazurin assay	-	EtOH (0.2%)	DNCB 5µg/mL	0.036	0.043	0.052	0.063	0.075	0.090
$FI_{mes.1}$	43401	43364	34974	38929	38050	36392	35889	34240	33455
$FI_{mes.2}$	43179	43152	35495	39383	37827	36915	34867	33926	32900
$FI_{mes.3}$	43417	42822	35043	39492	37610	36649	35223	34249	33432
Mean	43332	43113	35171	39268	37829	36652	35326	34138	33262
St. Dev.	133	273	283	299	220	262	519	184	314
Viability (% Control)									
Mean	100	100	80	90	87	84	81	78	75
St. Dev.	0.68	0.68	0.70	0.74	0.55	0.65	1.29	0.46	0.78
CD86 (%)									
Mean	100	100	770	212	292	434	494	534	565
St. Dev.	3.25	3.25	34.69	10.42	14.53	10.31	11.07	11.03	16.63

CD54 expression assay on THP1 cells – 24hrs treatment

	Control	Contr.$_{EtOH}$	PC	EO mg/mL					
CD54 assay	-	EtOH (0.2%)	DCNB 5μg/mL	0.036	0.043	0.052	0.063	0.075	0.090
OD$_{mes.1}$<br>OD$_{mes.2}$<br>OD$_{mes.3}$	0.279<br>0.262<br>0.268	0.252<br>0.280<br>0.264	0.794<br>0.819<br>0.793	0.552<br>0.540<br>0.538	0.567<br>0.585<br>0.582	0.590<br>0.637<br>0.597	0.671<br>0.701<br>0.681	0.700<br>0.707<br>0.711	0.712<br>0.711<br>0.702
Mean	0.270	0.265	0.802	0.543	0.578	0.608	0.684	0.706	0.708
St. Dev.	0.009	0.014	0.015	0.007	0.010	0.026	0.015	0.005	0.006
OD$_{CD54}$<br>Mean<br>St. Dev.	 0.614<br>0.036	 0.597<br>0.057	 2.743<br>0.060	 1.708<br>0.030	 1.848<br>0.039	 1.968<br>0.102	 2.272<br>0.062	 2.360<br>0.022	 2.368<br>0.023
Resazurin assay	-	EtOH (0.2%)	DNCB 5μg/mL	0.036	0.043	0.052	0.063	0.075	0.090
FI$_{mes.1}$<br>FI$_{mes.2}$<br>FI$_{mes.3}$	43401<br>43179<br>43417	43364<br>43152<br>42822	34974<br>35495<br>35043	38929<br>39383<br>39492	38050<br>37827<br>37610	36392<br>36915<br>36649	35889<br>34867<br>35223	34240<br>33926<br>34249	33455<br>32900<br>33432
Mean	43332	43113	35171	39268	37829	36652	35326	34138	33262
St. Dev.	133	273	283	299	220	262	519	184	314
Viability (% Control)<br>Mean<br>St. Dev.	 100<br>0.68	 100<br>0.68	 80<br>0.70	 90<br>0.74	 87<br>0.55	 84<br>0.65	 81<br>1.29	 78<br>0.46	 75<br>0.78
CD54 (%)<br>Mean<br>St. Dev.	 100<br>5.58	 100<br>9.69	 560<br>7.31	 316<br>8.12	 356<br>9.26	 392<br>17.51	 471<br>20.04	 509<br>5.92	 525<br>8.98

THP1 cell line reactivity control – CD86 expression assay on THP1 cells – 24hrs treatment

	Control	PC1	PC2
CD86 assay	-	DNCB	$NiSO_4$
OD$_{mes.1}$<br>OD$_{mes.2}$<br>OD$_{mes.3}$	0.187<br>0.198<br>0.187	0.870<br>0.883<br>0.863	0.788<br>0.805<br>0.785
Mean	0.191	0.873	0.793
St. Dev.	0.006	0.010	0.011
OD$_{CD86}$<br>Mean<br>St. Dev.	 0.320<br>0.025	 3.045<br>0.041	 2.729<br>0.043
Resazurin assay	-	DNCB	$NiSO_4$
FI$_{mes.1}$<br>FI$_{mes.2}$<br>FI$_{mes.3}$	42990<br>43385<br>42954	34704<br>34107<br>34143	36305<br>36812<br>36997
Mean	43113	34318	36705
St. Dev.	236	335	358
Viability (% Control)<br>Mean<br>St. Dev.	 100<br>0.59	 78<br>0.83	 84<br>0.89
CD86 (%)<br>Mean<br>St. Dev.	 100<br>7.33	 1219<br>22.80	 1015<br>18.24

	Control	NC1
CD86 assay	-	LA
$OD_{mes.1}$<br>$OD_{mes.2}$<br>$OD_{mes.3}$	0.187<br>0.198<br>0.187	0.210<br>0.241<br>0.209
Mean	0.191	0.220
St. Dev.	0.006	0.018
OD_{CD86}<br>Mean<br>St. Dev.	 0.320<br>0.025	 0.437<br>0.072
XTT assay	-	LA
$OD_{mes.1}$<br>$OD_{mes.2}$<br>$OD_{mes.3}$	0.527<br>0.559<br>0.537	0.541<br>0.544<br>0.556
Mean	0541	0.547
St. Dev.	0.016	0.008
Viability (% Control)<br>Mean<br>St. Dev.	 100<br>3.46	 102<br>1.75
CD86 (%)<br>Mean<br>St. Dev.	 100<br>4.70	 135<br>23.12

THP1 cell line reactivity control – CD54 expression assay on THP1 cells – 24hrs treatment

	Control	PC1	PC2
CD54 assay	-	DNCB	$NiSO_4$
$OD_{mes.1}$<br>$OD_{mes.2}$<br>$OD_{mes.3}$	0.238<br>0.216<br>0.224	0.739<br>0.723<br>0.663	0.894<br>0.854<br>0.893
Mean	0.226	0.708	0.880
St. Dev.	0.011	0.040	0.023
OD_{CD54}<br>Mean<br>St. Dev.	 0.471<br>0.045	 2.401<br>0.163	 3.088<br>0.091
Resazurin assay	-	DNCB	$NiSO_4$
$FI_{mes.1}$<br>$FI_{mes.2}$<br>$FI_{mes.3}$	42990<br>43385<br>42954	34704<br>34107<br>34143	36305<br>36812<br>36997
Mean	43113	34318	36705
St. Dev.	236	335	358
Viability (% Control)<br>Mean<br>St. Dev.	 100<br>0.59	 78<br>0.83	 84<br>0.89
CD54 (%)<br>Mean<br>St. Dev.	 100<br>9.94	 653<br>40.08	 780<br>26.74

	Control	NC1
CD54 assay	-	LA
$OD_{mes.1}$<br>$OD_{mes.2}$<br>$OD_{mes.3}$	0.238<br>0.216<br>0.224	0.182<br>0.174<br>0.223
Mean	0.226	0.193
St. Dev.	0.011	0.026

OD_{CD54}<br>Mean<br>St. Dev.	 0.471<br>0.045	 0.338<br>0.105
XTT assay	-	LA
$OD_{mes.1}$<br>$OD_{mes.2}$<br>$OD_{mes.3}$	0.527<br>0.559<br>0.537	0.541<br>0.544<br>0.556
Mean	0.541	0.547
St. Dev.	0.016	0.008
Viability (% Control)<br>Mean<br>St. Dev.	 100<br>3.46	 102<br>1.75
CD54 (%)<br>Mean<br>St. Dev.	 100<br>12.78	 70<br>20.50
